# Changes in diet quality and home food environment in preschool children following weight management

**DOI:** 10.1186/s12966-019-0777-6

**Published:** 2019-02-04

**Authors:** Shannon M. Robson, Melissa L. Ziegler, Mary Beth McCullough, Cathleen Odar Stough, Cynthia Zion, Stacey L. Simon, Richard F. Ittenbach, Lori J. Stark

**Affiliations:** 1 Department of Behavioral Health and Nutrition, University of Delaware, 26 N College Avenue, Newark, DE 19716 USA; 2 Biostatistics Core, College of Health Sciences, University of Delaware, 540 S. College Avenue, Newark, DE 19713 USA; 3 Psychology Department, Suffolk University, 73 Tremont Street, Boston, MA 02108 USA; 4 Department of Psychology, University of Cincinnati, 47 W Corry Blvd Edwards 1 Bldg Suite 4130, P.O. Box 210376, Cincinnati, OH 45219 USA; 5 Division of Behavioral Medicine and Clinical Psychology, Cincinnati Children’s Hospital Medical Center, 3333 Burnet Avenue MLC 3015, Cincinnati, OH 45229 USA; 6 Department of Pediatrics, Division of Pulmonary Medicine, University of Colorado Anschutz Medical Campus and Children’s Hospital Colorado, 13123 E 16th Ave B395 Aurora, Aurora, CO 80045 USA; 7 Department of Pediatrics, University of Cincinnati College of Medicine, 3230 Eden Ave, Cincinnati, OH 45267 USA; 8 Division of Biostatistics and Epidemiology, Cincinnati Children’s Hospital Medical Center, 3333 Burnet Avenue MLC 5041, Cincinnati, OH 45229 USA

**Keywords:** Family-based obesity treatment, Preschool nutrition, Home food environment, Healthy eating index

## Abstract

**Background:**

Family-based obesity treatment interventions can successfully reduce energy intake in preschoolers. An implicit goal of obesity treatment interventions is to improve diet quality, but diet quality has been less examined as a treatment outcome in studies of preschoolers. The purpose of this study was to conduct a secondary analysis comparing the change in diet quality and home food environment in preschoolers assigned to a behavioral family-based obesity intervention (LAUNCH), motivational interviewing (MI) condition, or standard care (STC) condition.

**Methods:**

Three 24-h dietary recalls were completed at baseline and 6-months and were analyzed using NDS-R software; diet quality was assessed using the Healthy Eating Index-2010 (HEI-2010). Availability of foods and beverages in the home was assessed through direct observation using the Home Health Environment tool that classifies foods and beverages as ‘red’ or ‘green’ based upon fat and sugar content. Repeated measures linear mixed effects models were used to examine changes in diet quality and home food environment between conditions (LAUNCH, MI, STC).

**Results:**

At 6-months, preschoolers in the LAUNCH condition had a higher HEI-2010 total score (62.8 ± 13.7) compared to preschoolers in the MI (54.7 ± 13.4, *P* = 0.022) and STC (55.8 ± 11.6, *P* = 0.046) conditions. Regarding the home food environment, families in LAUNCH had significantly less ‘red’ foods in their home at 6-months (12.5 ± 3.4 ‘red’ foods) compared to families in MI (14.0 ± 3.7 ‘red’ foods, *P* = 0.030), and STC (14.3 ± 3.4 ‘red’ foods, *P* = 0.006). There were no statistically significant differences across home food environments for number of ‘green’ foods.

**Conclusion:**

Family-based obesity treatment interventions for preschoolers can improve overall diet quality and alter the home food environment through reductions in ‘red’ foods.

**Trial registration:**

Clinicaltrials.gov, NCT01546727. Registered March 7, 2012.

## Background

Behavioral family-based obesity treatment interventions effectively intervene on energy balance behaviors to promote weight loss through energy intake reduction in children as young as 2–5 years old [1–3]. Behavioral family-based obesity treatments use behavior modification strategies to alter parent feeding and child eating and physical activity patterns [2, 4]. Weight loss outcomes for up to 10 years have been reported for school age children who engaged in behavioral family-based obesity treatment [5]. However, it is unclear whether diet quality is also improving (an implicit goal of obesity treatment) or if children are simply eating less, but maintaining the same poor quality of diet [6]. Only one study of school age children (7–11 years-old) examined diet quality using the Healthy Eating Index (HEI) 2005 in a 16-week family-based obesity treatment intervention [7]. The HEI is a measure of adherence to the Dietary Guidelines for Americans based upon a scale of 0–100, with 100 demonstrating perfect adherence [8, 9]. Scores of 50 or lower are considered to reflect a “poor” diet, scores 51–79 indicate the diet “needs improvement,” and scores of 80 or greater reflect a “good” diet [10]. Altman and colleagues [7] found total HEI-2005 score improved from 59.3 ± 8.8 at baseline to 74.5 ± 9.8 post-treatment, indicating an improvement in overall diet quality over the 16-week period. This change was independent of changes in energy balance behaviors (energy intake and minutes of physical activity) and was associated with a reduction in eating foods away from home.

As pediatric obesity tracks across the life course [11–13], the early childhood years are considered a critical time to intervene on energy balance behaviors, especially diet, as children are hypothesized to be more susceptible to change during the preschool years [14]. During the preschool years, changes to energy balance through a reduction in energy intake can reduce obesity, which may prevent excess weight tracking into later childhood and adulthood [15]. In addition to weight management, the establishment of healthy eating behaviors during early childhood is also important due to the relationship between poor diet quality and other chronic diseases [16].

The current study conducted a secondary analysis examining change in diet quality for preschoolers enrolled in a randomized clinical trial where participants were assigned to a clinic and home-based behavioral, family-based obesity treatment program (LAUNCH), motivational interviewing (MI), or a standard care (STC) condition. Research has previously demonstrated that preschoolers receiving LAUNCH displayed a greater BMI z-score reduction compared to MI and STC; however, changes in diet quality between these groups have yet to be explored [1]. We hypothesized that diet quality, measured using the HEI-2010, would improve in preschoolers in the LAUNCH condition as compared to MI or STC.

As individual dietary intake is influenced by the home food environment, particularly the availability and accessibility of foods and beverages [17], family-based obesity treatments often recommend modifying the home food environment by adding or removing foods to encourage or discourage the consumption of these foods. However, few studies have assessed the home food environment of children undergoing a family-based obesity behavioral intervention and, therefore, the current study also examined changes in the home food environment for preschoolers participating in the clinical trial. We hypothesized that the home food environment would improve in the LAUNCH group with fewer ‘red’ foods defined as higher fat, high sugar foods and greater ‘green’ foods defined as lower fat, lower sugar foods in the home at 6-months.

## Methods

### Participants

From March 2012 to June 2015, preschoolers with obesity (BMI ≥ 95th percentile for age and sex) [18] were recruited through medical chart reviews from 27 independent pediatric practices and referrals from seven practices in a unified health system. Eligible children had a well-child visit within the last year and lived within 50 miles of the medical center where intervention sessions were conducted. Children were excluded if they had a medical condition (e.g., Prader-Willi syndrome) or were taking a medication that impacted weight (e.g., steroids); were participating in another weight management program, the caregiver or child had a medical condition or disability that would prevent participation in the program and/or physical activity (e.g., developmental delay); or the child or caregiver was non-English speaking. A total of 1629 children were invited/referred to participate, and 229 completed informed consent and were screened for eligibility. One hundred sixty-seven children met eligibility criteria and were randomized to a study condition; 151 met intent-to-treat criteria and were included in this analysis. Additional details regarding recruitment and enrollment of participants have been provided elsewhere [1]. The study protocol was approved by the Institutional Review Board at Cincinnati Children’s Hospital Medical Center and was prospectively registered on clinical trials.gov (NCT01546727).

### Study design

This study is a secondary analysis of data from a randomized clinical trial comparing LAUNCH, MI, and STC. The study conditions are described in brief below and full details are provided elsewhere [19, 20].

### Intervention

#### Launch

LAUNCH is a 6-month, 18-session family-based obesity treatment program developed specifically for preschoolers that consisted of alternating clinical-based group sessions and individual home-based visits. Clinic sessions were 90-min and were led by a clinical psychologist, and home-based visits were 60-min and were led by a postdoctoral fellow in psychology or nutrition, providing an intervention dose of 23 h over the 6 months. Caregivers were instructed to reduce their child’s caloric intake to 1000 kcals/day, 1200 kcals/day, or 1400 kcals/day depending upon their sex and age as recommended by the American Heart Association [21] and engage in 60 min/day of moderate-to-vigorous physical activity. As is standard in family-based behavioral obesity treatment programs, parents with a BMI ≥ 25 kg/m^2^ were also instructed to reduce calorie intake and engage in moderate-to-vigorous physical activity. Caregivers tracked dietary intake daily for themselves and their child. To assist with meeting calorie goals, parents and children were provided with information and skills to encourage purchasing and consumption of more ‘green’ foods and less ‘red’ foods.

#### Motivational interviewing (MI)

MI consisted of four in-person visits that were 60 min in length and 14 phone sessions (median phone session length was 15 min) delivered at the same frequency as LAUNCH. Using the median phone session length MI provided a total intervention dose of 7.5 h. During an initial session with a pediatrician, parents were provided with publicly available materials from the “Let’s Go 5-2-1-0” program. Subsequent sessions were conducted by a licensed clinical psychologist and followed the tenets of MI related to caregiver concern about the child’s weight, diet, and/or physical activity behaviors. Caregivers were presented a menu of target dietary and physical activity behaviors (i.e. increasing fruit and vegetable intake, decreasing screen time). Sessions were caregiver-driven behavioral changes with the therapist supporting problem-solving or providing information as requested.

#### Standard care (STC)

Families randomized to STC did not receive any treatment (0 contact hours) through the current study beyond being informed their child met criteria for obesity during the recruitment process.

### Measures

#### Demographics

Caregivers completed a self-report questionnaire regarding family, caregiver, and child demographics.

#### Dietary intake

Three random 24-h dietary recalls (2 weekdays and 1 weekend day), using the multiple pass method were conducted by trained Registered Dietitians blinded to treatment condition. The first dietary recall was conducted in person at the baseline visit with the two subsequent dietary recalls completed over the phone at unannounced times. For children who attended childcare (72.2% at baseline), with parent permission the Registered Dietitian called the childcare center to assess dietary intake. If the childcare center could not be contacted directly parents were instructed to have the childcare providers record what the child ate with paper and pencil daily until the two recalls were completed. Dietary recalls were analyzed using Nutrition Data Systems for Research software (versions 2012–2016, Nutrition Coordinating Center, University of Minnesota) to obtain average daily energy intake and servings from food groups (fruits and vegetables, sugar sweetened beverages, sweet and salty snack foods). The HEI-2010 was used to assess dietary quality. The HEI-2010 is a measure of adherence to the 2010 Dietary Guidelines for Americans and is derived from 12 components, including 9 adequacy components (total fruit, whole fruit, total vegetables, greens and beans, whole grains, dairy, total protein foods, seafood and plant proteins, and fatty acids) and 3 moderation components (refined grains, sodium, and empty calories). Higher scores for each component represent better diet quality with moderation components being reverse scored. Total HEI-2010 score can theoretically range from 0 to 100, with 100 indicating perfect adherence. Given when data were collected HEI-2010 was used, which is similar to HEI-2005 and the new HEI-2015. All versions are reflective of the Dietary Guidelines for Americans at that time and include adequacy and moderation components, and used a density approach to set standards. Several differences do exist between HEI-2005 and HEI-2010, specifically related to the components in regards to how they were defined and/or the standards for minimum/maximum points, and have been outlined by the National Cancer Institute [22].

#### Home food environment

The availability of foods and beverages in the home was assessed using the Home Health Environment, a direct observation tool [23]. The availability (yes/no) of foods (candy; prepared baked goods; baking items; chips, pretzels, popcorn and rice cakes; crackers; fruit roll-ups or fruit snacks; granola bars, breakfast bars and toaster strudels; dried fruit; cereal; pasta mixes; bread; meat; frozen foods; ice cream or frozen desserts; cheese, yogurt and cottage cheese; syrups) and beverages (100% fruit juice; fruit drinks; regular sodas; diet sodas; sport drinks; powdered drinks; milk) was recorded. Availability (yes/no) of fresh, frozen, and canned fruits and vegetables was also recorded. Foods, beverages and canned fruits were coded as ‘green’ or ‘red’ foods based upon fat and sugar content. Foods/beverages were summed across categories to obtain a total frequency of ‘red’ foods/beverages and ‘green’ foods/beverages (including fresh, frozen and canned fruits and vegetables). Total frequency of fresh, frozen and canned fruits and vegetables was also analyzed. A trained observer blinded to the families’ treatment assignment conducted all assessments of the home food environment. Twenty-six percent of randomly selected homes had a second independent observer code the home food environment for inter-rater reliability (IRR) with excellent reliability for all food and beverage categories (IRR ≥ 0.95).

### Statistical analysis

Descriptive statistics were computed for the group as a whole as well as by condition. Measures of central tendency, variability, and association were computed for all relevant treatment-related variables. All tests were conducted using alpha = 0.05, and summary measures are presented as mean ± standard deviation. A repeated measures linear mixed effects model was used to examine changes in the primary outcome variables diet quality (as measured by HEI-2010 total score), and ‘red’ foods and ‘green’ foods in the home food environment, and secondary outcome dietary intake variables (energy intake, food group servings) and home food environment variables (fruits, vegetables, fruits and vegetables) across the two study time points (baseline and 6-months) comparing the three conditions (LAUNCH, MI and STC) accounting for the correlation between measures on the same subject by directly modeling the covariance structure of the residuals as unstructured. For energy intake, models were adjusted for age, sex, and baseline body weight. Tests for mean differences were conducted using the Kenward-Roger approximation for the degrees of freedom; *p*-values comparing the mean responses between time points were adjusted for multiple comparisons using the Tukey-Kramer method [24]. All statistical analyses were performed using SAS software, Version 9.4 (SAS Institute, Cary, NC).

## Results

Participants were 151 preschoolers (55.1 ± 11.2 months) who met criteria for obesity (BMI percentile: 98.6 ± 1.3; BMI z-score: 2.4 ± 0.6). Children were 57% female, predominantly White (76%) and non-Hispanic (94%) and participated with one designated caregiver, who were primarily mothers (90%). Caregivers were most likely to be married (72%), have a college degree (53%) and have obesity (67%) defined as a BMI ≥ 30 kg/m^2^. At baseline, demographics and anthropometrics were not significantly different between conditions. See Table 1 for complete participant demographics.

**Table 1:** Baseline demographic and anthropometrics characteristics of preschool children enrolled in a family-based obesity treatment intervention

	Overall *n* = 151	LAUNCH *n* = 47	MI *n* = 50	STC *n* = 54
Child Age (months), M ± SD	55.1 ± 11.2	55.1 ± 12.1	55.0 ± 10.7	55.3 ± 11.1
Child Sex *n* (%)				
Male	65 (43.0%)	22 (46.8%)	21 (42.0%)	22 (40.7%)
Female	86 (57.0%)	25 (53.2%)	29 (58.0%)	32 (59.3%)
Child Ethnicity *n* (%)				
Hispanic or Latino	9 (6.0%)	1 (2.1%)	3 (6.0%)	5 (9.3%)
Non-Hispanic	142 (94.0%)	46 (97.9%)	47 (94.0%)	49 (90.7%)
Child Race *n* (%)				
Black	14 (9.3%)	3 (6.4%)	6 (12.0%)	5 (9.3%)
White	115 (76.2%)	37 (78.7%)	38 (76.0%)	40 (74.1%)
More Than One Race/Other	22 (14.6%)	7 (14.9%)	6 (12.0%)	9 (16.7%)
Child BMI M ± SD	20.9 ± 2.5	20.9 ± 2.5	20.7 ± 2.4	21.0 ± 2.6
Child BMI Z-score M ± SD	2.44 ± 0.60	2.41 ± 0.53	2.41 ± 0.56	2.48 ± 0.70
Child BMI Percentile M ± SD	98.6 ± 1.3	98.6 ± 1.2	98.5 ± 1.3	98.6 ± 1.3
Family Income *n* (%)				
< $30 k	16 (10.6%)	4 (8.5%)	8 (16.0%)	4 (7.4%)
$30 k – 49.9 k	23 (15.2%)	9 (19.2%)	5 (10.0%)	9 (16.7%)
$50 k – 99.9 k	77 (51.0%)	25 (53.2%)	23 (46.0%)	29 (53.7%)
≥ $100 k	34 (22.5%)	9 (19.2%)	14 (28.0%)	11 (20.4%)
Not Reported	1 (0.7%)	0 (0.0%)	0 (0.0%)	1 (1.9%)
Hollingshead Score^1^ M ± SD	43.0 ± 11.7	43.2 ± 11.1	42.2 ± 12.7	43.6 ± 11.3

^1^The Hollingshead Four Factor Index of Social Status is calculated based upon the education, occupation, sex, and marital status of the head(s) of households. Computed scores range from 8 to 66 with higher scores indicating higher family social status as attributed by other members of society

### Dietary intake

There was a significant (*P* = 0.007) condition by time interaction for HEI-2010. At 6-months, HEI-2010 total score was significantly higher in LAUNCH (62.8 ± 13.7) as compared to MI (54.7 ± 13.4, *P* = 0.022), and STC (55.8 ± 11.6, *P* = 0.046). Overall, children had a HEI-2010 total score of 56.6 ± 10.7 at baseline (*n* = 151) and 57.7 ± 13.3 at 6-months (*n* = 135). Analyses for HEI component scores are shown in Table 2.

**Table 2:** Changes in energy intake, dietary quality and servings of food groups in preschool children enrolled in a family-based obesity treatment intervention^1^

	LAUNCH^1^	MI^1^	STC^1^	Condition^2^	Time^2^	Condition x Time^2^
Energy Intake^3^				0.058	< 0.001***	< 0.001***
Baseline	1455.6 ± 300.6	1425.0 ± 356.2	1323.0 ± 273.8			
6 months	1147.8 ± 216.5	1410.6 ± 333.6	1408.8 ± 337.1			
HEI-2010 total score [0–100]				0.147	0.212	0.007**
Baseline	56.2 ± 11.0	55.6 ± 9.7	57.9 ± 11.4			
6 months	62.8 ± 13.7	54.7 ± 13.4	55.8 ± 11.6			
Total Fruit [0–5]				0.245	0.972	0.094
Baseline	3.5 ± 1.6	3.2 ± 1.7	3.3 ± 1.9			
6 months	3.7 ± 1.7	3.3 ± 1.7	2.8 ± 1.6			
Whole Fruit [0–5]				0.234	0.210	0.236
Baseline	3.5 ± 1.8	3.7 ± 1.6	3.3 ± 1.9			
6 months	4.1 ± 1.4	3.7 ± 1.7	3.2 ± 1.7			
Total Vegetables [0–5]				0.051	0.045*	0.010*
Baseline	2.8 ± 1.5	2.8 ± 1.5	3.2 ± 1.5			
6 months	3.7 ± 1.5	2.6 ± 1.6	3.4 ± 1.4			
Greens and Beans [0–5]				0.770	0.002**	0.379
Baseline	0.5 ± 1.3	0.7 ± 1.4	0.6 ± 1.2			
6 months	1.4 ± 1.9	0.9 ± 1.6	0.9 ± 1.6			
Whole Grains [0–10]				0.422	0.026*	0.127
Baseline	3.7 ± 3.0	3.9 ± 2.9	3.4 ± 3.0			
6 months	5.1 ± 3.3	3.8 ± 3.4	4.0 ± 3.0			
Dairy [0–10]				0.045*	0.782	0.502
Baseline	9.4 ± 1.3	8.7 ± 2.1	8.6 ± 2.2			
6 months	9.3 ± 1.8	8.7 ± 2.2	8.9 ± 1.8			
Total Protein Foods [0–5]				0.458	0.758	0.003**
Baseline	3.6 ± 1.3	4.1 ± 1.3	4.0 ± 1.2			
6 months	4.2 ± 1.2	3.5 ± 1.4	4.1 ± 1.1			
Seafood and Plant Proteins [0–5]				0.997	0.785	0.027*
Baseline	1.3 ± 1.7	2.0 ± 2.1	2.0 ± 2.0			
6 months	2.2 ± 2.2	1.5 ± 1.9	1.4 ± 1.9			
Fatty Acids [0–10]				0.128	0.550	0.164
Baseline	3.4 ± 3.0	3.3 ± 3.0	4.0 ± 3.1			
6 months	4.2 ± 3.0	2.7 ± 2.2	3.2 ± 2.9			
Sodium [0–10]				0.132	0.073	< 0.001***
Baseline	4.4 ± 2.5	3.4 ± 2.7	4.8 ± 2.9			
6 months	2.6 ± 2.4	4.6 ± 3.1	3.8 ± 2.6			
Refined Grains [0–10]				0.694	0.118	0.333
Baseline	5.5 ± 3.5	6.2 ± 2.8	6.3 ± 3.2			
6 months	5.5 ± 3.6	5.2 ± 2.8	5.7 ± 3.2			
Empty Calories [0–20]				0.007**	0.022*	0.012*
Baseline	14.7 ± 4.1	13.7 ± 4.2	14.3 ± 3.5			
6 months	17.0 ± 3.3	14.0 ± 3.9	14.2 ± 4.3			
Servings of FVs				0.168	0.009**	0.119
Baseline	2.1 ± 1.4	2.0 ± 1.2	2.0 ± 1.2			
6 months	2.8 ± 1.7	2.4 ± 1.6	2.0 ± 1.0			
Servings of Fruits				0.012*	0.114	0.040*
Baseline	1.3 ± 1.1	1.2 ± 0.9	1.1 ± 0.9			
6 months	1.7 ± 1.2	1.5 ± 1.3	0.9 ± 0.7			
Servings of Vegetables				0.574	0.016*	0.524
Baseline	0.8 ± 0.6	0.8 ± 0.7	0.9 ± 0.7			
6 months	1.1 ± 0.8	0.9 ± 0.9	1.1 ± 0.8			
Servings of SSB				0.011*	0.022*	0.003**
Baseline	0.7 ± 0.7	1.0 ± 1.2	0.8 ± 0.7			
6 months	0.3 ± 0.4	0.7 ± 0.6	0.9 ± 1.0			
Servings of SSS				0.465	0.216	0.033*
Baseline	1.9 ± 1.3	1.8 ± 1.1	1.7 ± 1.0			
6 months	1.3 ± 0.8	1.9 ± 1.4	1.8 ± 1.4			

^1^Values are raw data means ± SDs. At baseline LAUNCH, *n* = 47; MI, *n* = 50; STC, *n* = 54 and at 6 months LAUNCH, *n* = 43; MI = 44; STC = 48. ^2^Values are *P*-values

^3^Energy intake is adjusted for sex, age, and baseline weight

HEI-2010 = Healthy Eating Index – 2010; FVs = fruits and vegetables; SSB = sugar sweetened beverages; SSS = sweet and salty snack foods

*P < 0.05; ** *P* < 0.01; ***P < 0.001

There was a significant (*P* < 0.001) condition by time interaction for energy intake. At 6-months LAUNCH (1147.8 ± 216.5 kcals) had a significantly lower mean energy intake as compared to MI (1410.6 ± 333.6 kcals, *P* < 0.001) and STC (1408.8 ± 337.1 kcals, *P* < 0.001). The reduction in energy intake corresponds with the main outcomes of the primary study demonstrated LAUNCH had a significantly greater decrease in BMI z-score (− 0.32 ± 0.33) as compared with MI (− 0.05 ± 0.27, *P* < 0.001) and compared with STC (− 0.13 ± 0.31, *P* < 0.004) [1].

There was not a significant time by condition interaction for consumption of servings of fruits and vegetables, but at 6-months children in LAUNCH consumed significantly more servings of fruits and vegetables (2.8 ± 1.7 servings) as compared to STC (2.0 ± 1.0 servings, *P* = 0.040). When servings of fruits and vegetables were examined separately there was a significant (*P* = 0.040) condition by time interaction for fruit, but not for vegetables. At 6-months children in LAUNCH consumed significantly more servings of fruit (1.7 ± 1.2 servings) as compared to STC (0.9 ± 0.7 servings, *P* < 0.001), but not MI (1.5 ± 1.3 servings, *P* = 0.366). Sugar sweetened beverage intake and sweet and salty snack foods also had a significant time by condition interactions. At 6-months children in LAUNCH consumed significantly fewer servings of sugar sweetened beverages (0.3 ± 0.4 servings) as compared to MI (0.7 ± 0.6 servings, *P* = 0.005), and STC (0.9 ± 1.0 servings, *P* < 0.001). Similarly, children in LAUNCH consumed fewer servings (1.3 ± 0.8 servings) of sweet and salty snack foods at 6-months as compared to MI (1.9 ± 1.4 servings, *P* = 0.040), but not STC (1.8 ± 1.4 servings, *P* = 0.131).

### Home food environment

As shown in Table 3, across all conditions there was a significant condition by time interaction (*P* < 0.001) for ‘red’ foods in the home. At 6-months, families in LAUNCH had significantly less ‘red’ foods in their home (12.5 ± 3.4 ‘red’ foods) as compared to families in MI (14.0 ± 3.7 ‘red’ foods, *P* = 0.030) and STC (14.3 ± 3.4 ‘red’ foods, *P* = 0.006). There was not a significant main effect of time, condition or a significant time by condition interaction for ‘green’ foods, fruits, or vegetables.

**Table 3:** Frequency of foods in the home food environment of preschool children with obesity enrolled in a family-based obesity treatment intervention

	LAUNCH^1^	MI^1^	STC^1^	Condition^2^	Time^2^	Condition x Time^2^
‘Red’ Foods				0.170	< 0.001***	< 0.001***
Baseline	15.3 ± 3.4	14.4 ± 4.7	15.6 ± 3.4			
6 months	12.5 ± 3.4	14.0 ± 3.7	14.3 ± 3.4			
‘Green’ Foods				0.340	0.495	0.069
Baseline	24.6 ± 8.3	23.8 ± 8.2	25.4 ± 7.2			
6 months	26.8 ± 8.1	22.8 ± 7.7	24.2 ± 8.1			
Fruits				0.800	0.443	0.603
Baseline	6.0 ± 3.0	5.9 ± 3.0	5.9 ± 3.2			
6 months	6.4 ± 3.0	5.6 ± 3.2	5.8 ± 3.3			
Vegetables				0.407	0.330	0.123
Baseline	10.6 ± 4.6	10.2 ± 4.4	10.8 ± 4.8			
6 months	11.5 ± 4.6	9.4 ± 4.0	10.2 ± 4.8			
FVs				0.500	0.234	0.148
Baseline	16.6 ± 6.7	16.2 ± 6.3	16.7 ± 6.6			
6 months	17.8 ± 6.9	15.0 ± 5.9	15.9 ± 7.2			

^1^Values are raw data means ± SDs. At baseline LAUNCH, *n* = 47; MI, *n* = 50; STC, *n* = 54 and at 6 months LAUNCH, *n* = 43; MI = 44; STC = 48. ^2^Values are *P*-values

FVs = fruits and vegetables

*P < 0.05; ** *P* < 0.01; ***P < 0.001

## Discussion

LAUNCH, a behavioral family-based obesity treatment delivered through clinic and home-based sessions, resulted in lower energy intake and also higher overall diet quality at 6-months as compared to MI and STC conditions. Consistent with the literature reporting the poor quality of preschool children’s diet, preschool children enrolled in this study had overall diet quality at baseline that needed improvement with a mean HEI total score of 56.6 ± 10.7 mirroring scores reported for preschool-aged children nationally 55.3 (95% CI: 53.6, 57.0) [6]. While children receiving LAUNCH had a statistically significant improvement in mean HEI-2010 total score from 56.2 ± 11.0 at baseline to 62.8 ± 13.7 at 6-months, their overall diet quality as measured by HEI continued to be in the “needs improvement” category (scores of 51–79) [10]. The ability of behavioral family-based obesity treatment programs to reduce energy intake and improve overall diet quality is promising and may provide benefits for chronic disease prevention beyond obesity. While chronic disease associated with poor dietary intake is not often present in preschool-aged children due to the limited time for disease progression, in adults higher diet quality scores have been associated with lower risk of cardiovascular disease and cancer [25–27].

In preschool children, changes to specific food groups (e.g. fruits and vegetables) have not been found to significantly predict changes in BMI z-score [15]. However, data from several studies indicate a reduction in higher calorie, lower nutrient (‘red’) foods appear to be a stronger underlying mechanism of weight change outcomes as compared to changes in lower calorie, higher nutrient (‘green’) foods. In 7–12 year-old children participating in a family-based obesity treatment, Best and colleagues [28] found long-term change in self-reported ‘red’ food intake predicted children’s weight loss maintenance while change in fruits and vegetables did not. Data from the current study support this notion as changes in categories associated with higher calorie, lower nutrient dietary intake (e.g. discretionary calories, servings of sugar sweetened beverages, servings of sweet and salty snack foods, ‘red’ foods) were significant while changes in lower calorie, higher nutrient foods (e.g. fruits and vegetables, ‘green’ foods) were not.

While reductions in high calorie, low nutrient foods may be more strongly associated with reductions in energy intake, it appears these foods are not replaced with high nutrient, low calorie foods [29, 30]. Golley and colleagues [31] found a reduction in high calorie, low nutrient foods in 6–9 year-old children participating in a 6-month weight management program in Australia, but intake of fruits and vegetables was unchanged. While this specific relationship was not analyzed, children enrolled in LAUNCH had significant dietary changes across high calorie, low nutrient (e.g. sweet and salty snack foods, sugar sweetened beverages) and low calories, high nutrient food groups (e.g. fruits and vegetables), but only when compared to STC and not when compared to MI. Individual dietary intake changes were small, in the amount of approximately a ¼-cup equivalent; however, they were adequate enough to reduce energy intake and improve dietary quality.

In addition to changes in child dietary intake, we found similar findings in the home food environment. The home environment has been identified as an important mediator of improved outcomes during family-based obesity treatment [32]. Study pilot data demonstrated that a family-based obesity treatment with home visitation resulted in significant increases in available servings of fruits and vegetables and decreased high-calorie foods in the home food environment as compared to pediatric counseling [33]. The LAUNCH condition, which included home visitation, similarly found a significant reduction in ‘red’ foods; however, in contrast to our pilot study, availability of fruits and vegetables did not increase significantly despite an increase in consumption of servings in the LAUNCH condition and decreases in the MI and STC condition.

The current study has several strengths, particularly the strong assessment measures including multiple 24-h recalls conducted by Registered Dietitians blinded to treatment assignment and the use of direct observation home food environment assessment based upon open inventory methodology where all foods and beverages available are recorded. Assessing data at both the individual and home food environment level may strengthen findings related to dietary outcomes, but further investigation would be needed to evaluate concordance between measures. Findings need to be interpreted within the context of study limitations. Intervention dose between conditions was different as designed given the primary study specifically compared LAUNCH to STC and LAUNCH to MI. Given this study compared between conditions, findings in the LAUNCH condition could be influenced by the greater intervention dose received by families as compared to MI and STC. Dietary data were self-reported by caregivers of children, and may be limited to the recall memory of caregiver, but also be subject to self-report bias. While the Home Food Environment tool has not been validated it was been previously used [23] and 26% of homes had a second independent observer to demonstrate inter-rater reliability. Furthermore, the sample was treatment-seeking families of preschoolers with obesity from primarily White, high-income, two-parent households, which may limit generalizability to other populations.

## Conclusions

As hypothesized LAUNCH had a greater improvement in diet quality as measured by HEI-2010 score, and a greater decrease in ‘red’ foods in the home food environment as compared to MI and STC. However, contrary to our hypothesis there was not a significant increase in ‘green’ foods in the home food environment in the LAUNCH condition. While the improvement in diet quality was encouraging, these changes did not move the children into the “good” range for diet quality on the HEI; this may indicate that additional attention should be focused on diet quality in the context of weight management programs for preschoolers. To have an impact on diet quality and the home food environment outcomes from this study suggest a stronger focus on reducing high calorie, low nutrient foods (e.g. SSBs, SSS) instead of increasing lower calorie, higher nutrient foods (e.g. fruits, vegetables) will be most beneficial.

## Abbreviations

BMI: Body Mass Index
HEI: Healthy Eating Index
LAUNCH: a clinic and home-based behavioral, family-based obesity treatment program
MI: Motivational interviewing intervention
STC: Standard of care
